# Incidental diagnosis of pseudomyxoma peritonei associated with peritoneocele at MRI defecography: a case report

**DOI:** 10.3389/fonc.2026.1727860

**Published:** 2026-05-08

**Authors:** Laura Maria Chisari, Giorgio La Greca, Gaetano Valenti, Fabio Ciancio, Cecilia Gozzo

**Affiliations:** 1 Department of Radiology, Humanitas Centro Catanese di Oncologia, Catania, Italy; 2 Department of Coloproctology and Pelvic Floor Surgery, Istituto di Ricovero e Cura a Carattere Scientifico (IRCCS) Policlinico San Donato, San Donato Milanese, Italy; 3 Department of Gynecology, Humanitas Centro Catanese di Oncologia, Catania, Italy; 4 Department of Biomedical Science, Humanitas University, Milan, Italy

**Keywords:** ascites, defecography, MRI defecography, pelvic floor disorders, pseudomyxoma peritonei, pelvic tumor

## Abstract

In the setting of pelvic disorders, before the introduction of MRI defecography, conventional fluoroscopic defecography was the gold standard. Compared to this latter, MRI defecography has the advantage of providing a full pelvic study, enabling the diagnosis of any coexisting diseases (i.e. oncological or inflammatory ones) that could be missed on conventional fluoroscopic defecography. We present an incidental diagnosis of pseudomyxoma peritonei causing a peritoneocele detected at MRI defecography. A 60-year-old healthy woman came to a proctology consultation complaining a sensation of pelvic heaviness over the past three months. Proctological examination revealed a soft bulge of tissue in the vagina suspect for enterocele to be included in differential diagnosis with peritoneocele. If a conventional defecography had been performed, in place of MRI defecography, it would show neither a peritoneocele nor the mucinous ascites, delaying its detection and underestimating the severity of the disease. In this clinical case, the choice to perform the MRI defecography instead of conventional defecography has been crucial in diagnosing a severe oncological disease allowing a consequent prompt treatment. This highlights the important role of MRI defecography in the global assessment of pelvic disorders, due to the ability to detect other pelvic pathological conditions.

## Introduction

In the setting of pelvic disorders, before the introduction of MRI defecography (MRD), conventional fluoroscopic defecography (FD) was the gold standard. Compared to the latter, MRD has the advantage of providing a full pelvic study, enabling the detection of any coexisting diseases (i.e., oncological or inflammatory ones) that could be missed on FD (1, 2).

Regarding oncological diseases, advanced pseudomyxoma peritonei (PMP) can occur with progressive abdominal distention causing pelvic disorders (i.e., cul-de-sac hernias) (3, 4). Among the latter, peritoneocele is the herniation of peritoneal fold into the rectovaginal space, appearing as a rectovaginal septum bulge (5).

PMP is a neoplastic condition characterized by mucin dissemination within the peritoneal cavity, typically related to appendiceal mucinous neoplasia (AMN) and rarely to a heterogeneous group of mucin-producing epithelial neoplasms such as ovarian or gastrointestinal cancer (6–8).

The combination of complete cytoreductive surgery (CRS) followed by hyperthermic intraperitoneal chemotherapy (HIPEC) improves patient survival (9–11).

Herein, we present an incidental diagnosis of PMP associated with peritoneocele at MRD.

## Case description

A 60-year-old healthy woman came to a proctology consultation complaining a sensation of pelvic heaviness over the past 3 months. She denied gastrointestinal symptoms such as vomiting, diarrhea, constipation, or weight loss. Proctological examination revealed a soft bulge of tissue in the vagina suspect for enterocele.

**Figure 1:**
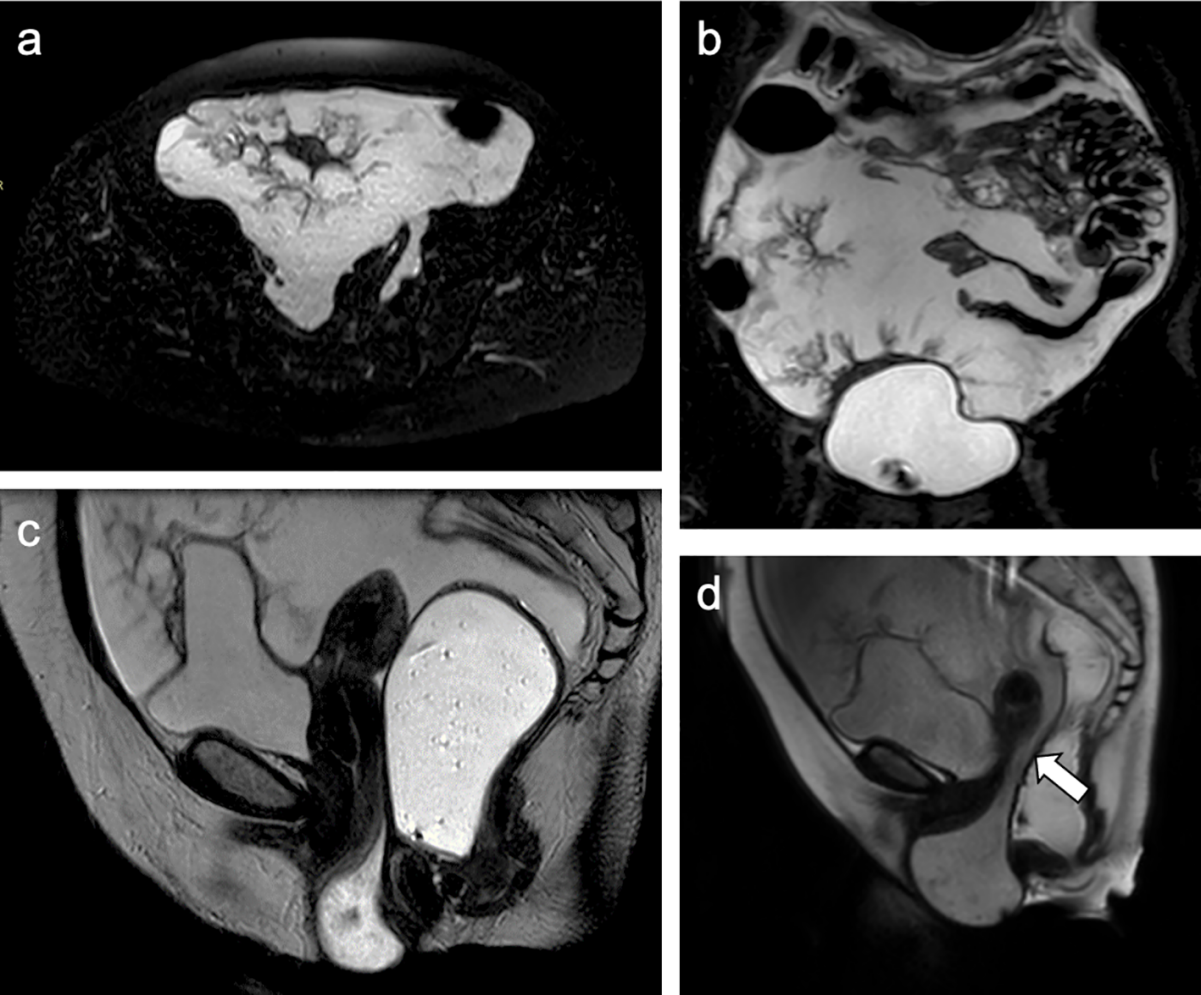
Axial **(a)** and coronal **(b)** T2W sequence showed a large amount of hyperintense fluid of mucinous nature with peritoneal nodules; sagittal **(c)** T2W sequence during the resting phase showed a large prolapse containing fluid in the rectovaginal space; sagittal **(d)** T2W during the evacuation phase showed a communication between the peritoneal mucinous fluid and the prolapse, suspected for peritoneocele.

In order to study pelvic floor dysfunction, an MRD was performed. **Table 1** summarizes the MRI technique. At MRD, the first axial and sagittal T2-weighted sequences showed a large volume of mucinous free fluid with peritoneal nodules predominantly near the cecum and bladder (**Figures 1a–c**). At the resting phase, in the rectovaginal space, a large prolapse containing fluid was confirmed; the evacuation phase showed a communication between the peritoneal mucinous fluid and the prolapse, excluding the suspicion of enterocele and diagnosing a peritoneocele (**Figure 1d**). These MRI findings led us to suspect the presence of PMP causing peritoneocele.

**Table 1:** Defecography-MRI protocol.

MRI sequence	Imaging plane
T2W TSE HR	Axial
T1W TSE	Axial
T2W TSE HR	Coronal
T2W TSE HR	Sagittal
T2W SSH-TSE	Midsagittal, dynamic at rest
T2W SSH-TSE	Midsagittal, dynamic at defecation
T2W SSH-TSE	Midsagittal, dynamic at squeezing
T2W SSH-TSE	Midsagittal, dynamic at Valsalva maneuver

T2W, T2 weighted; TSE, Turbo Spin Echo; HR, High Resolution; T1W, weighted; SSH, Single-Shot Turbo Spin-Echo.

**Figure 2:**
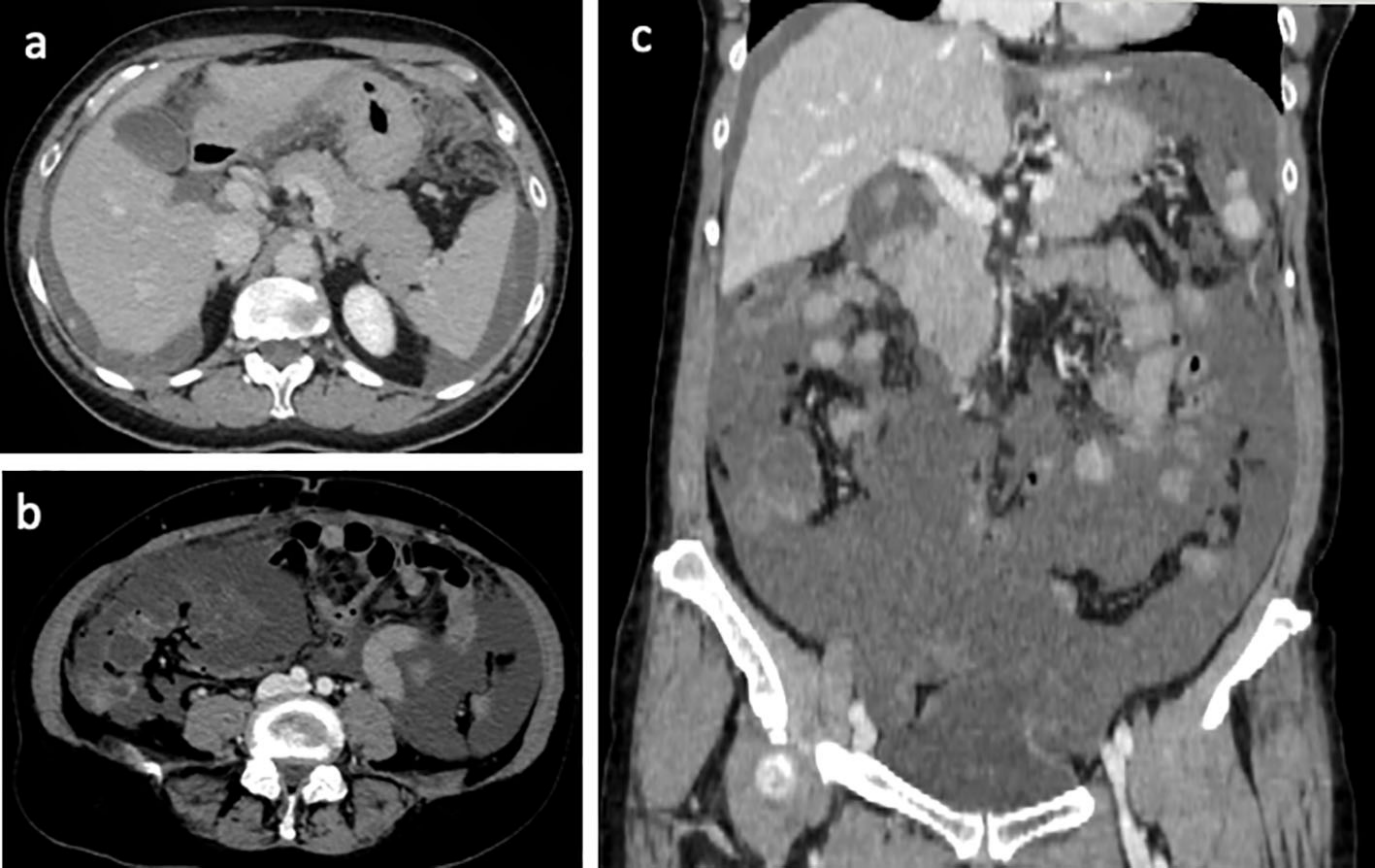
Axial **(a, b)** and coronal **(c)** portal CT scan showed ascites with liver scalloping and peritoneal implants.

In order to stage the disease, a thoraco-abdominal contrast-enhanced computed tomography (CT) scan was performed. CT scan confirmed ascites with liver scalloping and peritoneal implants (**Figure 2**). No distant metastases were detected. These CT findings strongly suggested PMP, likely of appendiceal or ovarian origin.

**Figure 3:**
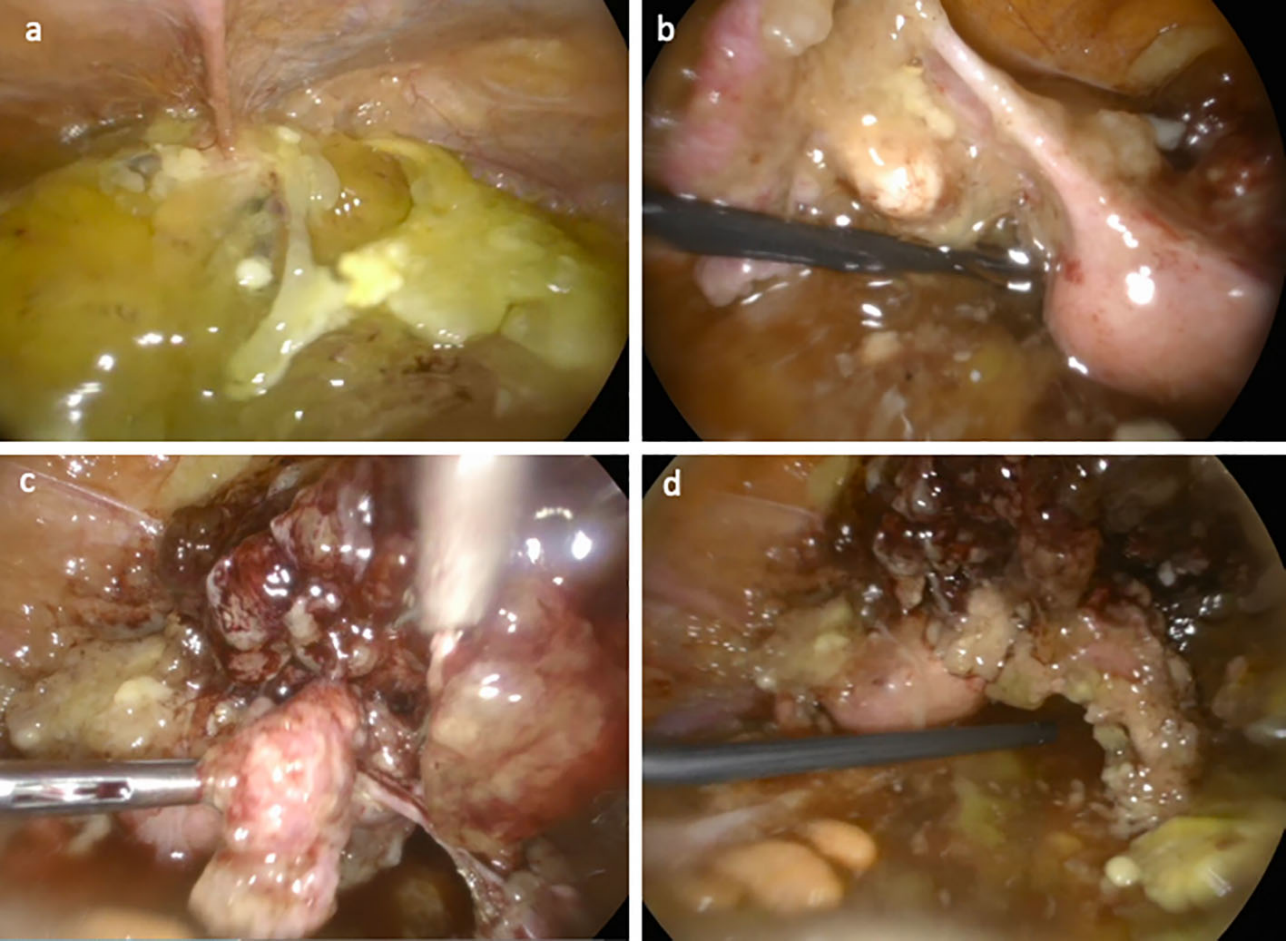
Intraoperative laparoscopic imaging show the following: **(a)** the peritoneal cavity filled with mucinous-like material **(b)** once the uterus appears elevated, with the round ligament pulled upward, the left ovarian fossa is occupied by mucinous-like material, which envelops the ipsilateral adnexa; **(c)** the right fallopian tube covered by a cerebriform mucinous lesion, originating from the appendix; and **(d)** an overview of the cerebriform mucinous lesion extending from the appendix, enveloping the right adnexa and the anterior surface of the uterus.

As part of laboratory findings, tumor marker analysis showed a carcinoembryonic antigen (CEA) level of 32.55 ng/mL (normal range: 0.00–5.00 ng/mL) and a cancer antigen 125 (CA-125) level of 73.20 U/mL (normal range: 0.00–35.00 U/mL), strengthening the suspicion of an underlying neoplastic process. In order to exclude gynecological cancers, a transvaginal ultrasound (US) showed uterus and adnexa of normal shape and size consistent with a postmenopausal woman; it also revealed a pelvis completely occupied by low-level echogenic areas with trabeculated regions, suggestive of compartmentalized mucin. Similar findings were observed in the paracolic gutters, Morrison’s pouch, and perisplenic space. The patient underwent diagnostic exploratory laparoscopy (**Figure 3**), which showed mucinous ascitic fluid and peritoneal implants in the right iliac fossa, adherent to the cecum. Consequently, a peritoneal lavage, evacuation of 45 mL of mucinous ascites, and peritoneal biopsy for definitive histopathological evaluation were carried out.

Histological results showed a low-grade appendiceal mucinous neoplasm (LAMN).

The patient was immediately referred for CRS followed by HIPEC.

## Discussion

MRD, typically used for pelvic floor disorders, has some advantages compared to FD: most of all, it allows a global pelvic evaluation without exposure to ionizing radiation (2).

In this clinical case, the first MRI sequences had already revealed a large volume of mucinous free fluid and dynamic sequences, confirming its correlation with pelvic floor disorders (i.e., peritoneocele).

The latter, once mucinous ascites is detected, advanced the hypothesis of an oncological cause (i.e., PMP).

PMP is a rare clinical entity, more commonly observed in women aged 50–59, usually diagnosed on imaging for unrelated pathology or as an incidental finding at laparoscopy/laparotomy for an acute abdomen (9, 10). Early PMP is usually asymptomatic, while advanced disease presents with progressive abdominal distention, ascites, hernias, and pelvic pressure (3, 4, 9). Regarding the prognosis, the introduction of CRS and HIPEC significantly improved the survival of patients with PMP, showing a median 5- and 10-year overall survival of 87.4% and 70.3%, respectively (4, 9–11).

If a conventional FD had been performed, in place of MRD, it would show neither a peritoneocele nor mucinous ascites, delaying its detection and underestimating the severity of the disease.

In fact, a limitation of a conventional FD is its inability to differentiate peritoneocele from enterocele, unless adding contrast media administration in vagina and small bowel (12). Conversely, MRD is the only method that quantifies peritoneocele (13).

As regards mucinous ascites, it is true that even an abdominal US would have shown peritoneal free fluid, but in an asymptomatic woman, complaining of only vaginal heaviness, it was right to focus on pelvic imaging. However, in suspected enterocele, the proctologist should consider differential diagnosis with peritoneocele, which could be missed at FD.

This case report suggests that MRD could be the best approach in evaluating pelvic floor disorder, owing to its ability to exclude other concomitant diseases.

## Conclusion

MRD is a dynamic imaging modality utilized for assessing pelvic floor dysfunctions with its expanding application due to the advantage of being less invasive and not requiring ionizing radiation.

In this clinical case, the choice to perform MRD instead of the conventional FD has been crucial in diagnosing a severe oncological disease, allowing a consequent prompt treatment.

Especially in suspected enterocele, the proctologist should consider differential diagnosis with peritoneocele, which may be related to an underlying oncological disease and could be excluded by MRD.

This highlights the important role of MRD in the global assessment of pelvic disorders, because of its ability to detect other pelvic pathological conditions.

## Data Availability

The original contributions presented in the study are included in the article, Further inquiries can be directed to the corresponding author/s.
